# Development and validation of a post-ICU syndrome in family caregivers scale (PICS-F Scale)

**DOI:** 10.1186/s12912-026-05207-5

**Published:** 2026-08-08

**Authors:** Jia Lei, Xiao Cheng, Ya Hong Cui, Yu Jiao, Lin Yang, Xin Yuan Tang, Lei Li

**Affiliations:** 1 School of Nursing, Shandong First Medcial University & Shandong Academy of Medical Sciences, No. 619 Changcheng Road, Tai’an, 271000 Shandong China; 2 Weifang People’s Hospital, Weifang, Shandong China; 3 The Second Affiliated Hospital of Shandong First Medical University, No. 366 Taishan Street, Tai’an, Shandong 271000 China

**Keywords:** Caregivers, Intensive care units, PICS-F, Psychometrics, Reproducibility of results, Surveys and questionnaires

## Abstract

**Background:**

Recent studies indicate that rapid advances in medical technology have led to a steady increase in intensive care unit (ICU) survivors. The stressful experience of admission can lead to a series of symptoms in family caregivers of ICU patients, which is referred as Post-Intensive Care Syndrome‑Family (PICS‑F). However, existing tools are single-domain assessments and can only cover a portion of the four main domains of PICS-F. A comprehensive evaluation requires the combined use of multiple scales, which may make the process complex and time-consuming. Therefore, this study aims to develop and validate the PICS‑F Scale as an early screening tool for the risk of PICS‑F that covers physical, mental, cognitive, and social domains in family caregivers of adult ICU survivors.

**Methods:**

The initial item pool was generated through a literature review. Two rounds of the modified Delphi survey were conducted by a multidisciplinary panel of experts. Psychometric evaluation was conducted using data from 468 family caregivers of ICU survivors who were recruited from two general hospitals in Shandong Province, China, from March to May 2025. Validity was assessed via exploratory factor analysis (EFA) and confirmatory factor analysis (CFA). Cronbach’s α was used to assess the internal consistency. Criterion validity was assessed by calculating the correlation coefficient with the 36-Item Short Form Health Survey questionnaire (SF-36) used as the criterion measure.

**Results:**

The final PICS-F Scale contains 22 items. EFA revealed a four-factor structure, corresponding to physical, mental, cognitive, and social dimensions, which explained 76.33% of the total variance. CFA showed satisfactory fit indices (SRMR = 0.075, RMSEA = 0.090, TLI = 0.917, and CFI = 0.927). The scale showed great internal consistency (Cronbach’s α = 0.934; subscales: 0.891–0.958). With respect to criterion validity, the correlation between the PICS-F Scale and SF-36 was *r* = -0.723 (*p* < 0.001).

**Conclusions:**

The scale is a valid and reliable early screening tool for rapidly identifying family members at high risk of PICS‑F in Chinese language settings. It can be used in the early phase after ICU survivors are transferred out of the ICU.

**Supplementary Information:**

The online version contains supplementary material available at https://doi.org/10.1186/s12912-026-05207-5.

## Background

Research indicates that in recent decades, the survival rates of ICU patients have been steadily increasing [1–3]. However, for many ICU patients and their family caregivers, the ICU experience can be traumatic, causing significant mental and emotional distress [4–6]. A study in 2025 revealed that ICU experience could greatly affect patients’ families, as 45.2% of relatives developed PICS-F, 36.5% exhibited depressive symptoms, 31.7% reported anxiety, and 24.0% showed PTSD [7].Thus, the Society of Critical Care Medicine formally designated the cluster of complications experienced by families after critical illness as Post-Intensive Care Syndrome-Family (PICS-F) in 2010 [8].In addition, PICS-F is not limited to families of patients with prolonged ICU stays. Even short-term admissions can trigger serious psychological impairment. As early as one week after ICU admission, 42%–66% of relatives develop anxiety and 14.6%–66.7% experience depression [9]. Thus, PICS-F should be identified and managed early [10].

PICS‑F refers to the increased risk of severe symptoms among family caregivers due to the profound stress caused by a relative’s ICU admission [11]. It manifests across multiple dimensions. Mental symptoms such as anxiety, depression, PTSD, and complicated grief are quite common [12–13]. Research suggests that 20% to 60% of family caregivers suffer from at least one of these symptoms [14], and anxiety, depression, as well as PTSD appear most frequently [15]. Physical symptoms such as sleep disturbances, headaches, loss of appetite, and fatigue can also trouble family caregivers [16]. Over time, cognitive impairment may develop, reducing decision-making abilities of family caregivers [17]. At the same time, the care of ICU patients may affect family caregivers’ social functions, having a negative impact on their social skills and interpersonal relationships [18]. These symptoms not only harm caregivers’ own health but also limit their ability to care for patients, and ultimately harm patient recovery [19].

Currently, there are no standardized criteria or consensus for the assessment and diagnosis of PICS-F. A systematic review revealed that a number of studies have used various distinct screening instruments to assess symptoms of PICS-F, such as the Beck Depression Inventory [20], Patient Health Questionnaire‑9 [21], Generalized Anxiety Disorder Scale [22], Primary Care PTSD Screen [23], and Epworth Sleepiness Scale [24], which limits the comparability of their findings. Moreover, as these instruments are not PICS-F-specific, key influencing factors may be omitted during assessment [25].

An ideal assessment tool for PICS-F should contain four domains of physical, mental, cognitive, and social aspects to fully evaluate a series of complex symptoms [17, 26]. However, few studies have examined the whole traumatic experiences of ICU patients’ family caregivers. Indeed, most of them are limited to assessing only a single dimension of the PICS-F. A narrative review identified only two tools, neither of them multidimensional [27]. The CAESAR tool, for example, is targeted at bereaved families, assessing their psychological distress and its long-term outcomes within a social framework [28]. While the Functional Assessment of Cancer Therapy-Cognitive Function questionnaire (FACT-Cog) only focuses on cognitive function [29].

A survey from North Carolina revealed that 84% of physicians reported needing more education on the PICS/PICS-F, and 91% deemed a checklist of common issues beneficial [30]. Previous studies have also shown considerable variation in the timing and frequency of PICS-F assessments. This inconsistency, largely due to the absence of a standardized instrument, limits the comparability and synthesis of clinical findings [31]. Therefore, there is a clear need for a valid and easily administered self-report tool that requires no specialized training or equipment and can comprehensively assess the four core dimensions of PICS-F in family caregivers of adult ICU survivors.

Given that existing tools are limited to single-domain assessment, require a mix of tests, or fail to cover all four dimensions of PICS-F, this study aimed to develop and validate the PICS-F Scale as an early screening tool to identify the risk of PICS-F across the physical, mental, cognitive, and social domains in family caregivers of adult ICU survivors.

## Methods

### Study design

The purpose of this study was to develop and validate the PICS-F Scale as an early screening tool for PICS-F risk, covering physical, mental, cognitive, and social domains in family caregivers of adult ICU survivors. It is a methodological study combining literature review, modified Delphi expert consultation, and psychometric validation. The scale was developed and tested in Chinese.

### Generation of the initial item pool

A comprehensive literature review was conducted and served as the methodological framework for the main symptom identification and the initial item pool generation. For this literature review, a research team of five members was formed, which included two associate professors, one head nurse specialized in critical care, and two master’s students. The inclusion criteria for this review were that the study involved primary caregivers of patients discharged from the ICU, that the caregivers were at least 18 years old, and that the main content covered potential changes in family caregivers in the physical, mental, cognitive, and social domains during the care process. The exclusion criteria were duplicate publications and articles for which the full text could not be obtained.

Search terms such as “caregiver,” “carer,” “care giver,” “post intensive care syndrome-family,” “PICS-F,” and “PICS-family” were used. Literature searches were conducted in CNKI, VIP, CBM, Wanfang, Web of Science, Embase, and PubMed, as well as the Cochrane Library. The literature search had no restrictions of publication date and included titles and abstracts across available databases from their inception to the present. Only publications in Chinese or English were considered. To ensure a comprehensive review, the reference lists of included studies were examined. We extracted descriptions of PICS‑F symptoms from the eligible studies and grouped them into domains to form the initial item pool. In the beginning, the scale’s instructions clarified its purpose of assessing experiences of a family member’s ICU admission, to assist participants in responding based on their feelings and situations over the past week.

### Delphi survey

The modified Delphi method was implemented to the greatest extent under the study’s constraints, with attention to its main principles of authority and representativeness in expert selection [32]. By purposive sampling, experts from general hospitals in Shandong Province were invited to this consultation. Eligibility criteria for experts included a minimum of ten years of specialized clinical experience with current engagement in critical care, nursing, or psychological practice, a bachelor’s degree or higher, a professional level of intermediate clinical specialist or higher, willingness to participate, and availability to take part in the study.

The Delphi consultations were planned to be conducted in two rounds from January to March 2025. The questionnaires were distributed by email or WeChat, with a 7‑day response window per round. The questionnaire consisted of an introduction to the study, a section for collecting demographic information, the preliminary PICS-F Scale items, and an assessment of the experts’ authority level. The second-round questionnaire added detailed guidelines for scoring the scale items. After the first round, expert feedback was collected and analysed, and the items were revised to form the second-round questionnaire. We planned to continue the Delphi process until all predefined criteria were met. These criteria included a response rate of ≥ 80% per round, an expert authority coefficient (Cr) ≥ 0.70, a statistically significant Kendall’s W (*p* < 0.05), and less than 10% of items requiring modification in a round. Once these criteria were satisfied, we would consider that expert consensus had been reached and the scale would be finalized. If the criteria were not met after a round, additional rounds would be conducted until they were satisfied.

Data were dual-entered into Excel and analysed using SPSS version 27.0 (IBM Corp., Armonk, NY, USA). The consistency of expert ratings was assessed with Kendall’s W. Engagement was reflected by the valid return rate. Expert authority was measured through the authority coefficient (Cr). It was calculated as (Cs + Ca)/2. Cs was the familiarity with the topic (rated as 1.0, 0.8, 0.6, 0.4, or 0.2, from “very familiar” to “not familiar at all”), and Ca was the self-rated judgment basis (range 0–1). A Cr value of 0.70 or higher indicated good authority [33]. Item retention was based on two criteria. First, items were removed if the mean importance score was < 4.0, if the coefficient of variation was > 0.25, or if fewer than 80% of the experts rated it ≥ 4. Second, any expert-suggested deletions prompted a full-panel review. The research team made the final decision on the basis of the feedback.

### Pre‑survey evaluation

A pilot test and a focus group session were conducted before the main survey in March 2025. Convenience sampling was used to invite ten family members of ICU patients to evaluate the readability and comprehensibility of the scale items. The whole process lasted thirty minutes. First, the participants filled out the scale. After that, we asked them whether the item wording was clear and easy to understand, whether the scope of the assessment was comprehensive, and whether the scale was usable. The results showed that the family members could correctly understand the items, and no major revisions were suggested.

### Participant selection

A formal survey was conducted after the pilot test at two general hospitals in Shandong Province, China, from March to May 2025. A convenience sampling method was used to recruit participants. Family caregivers in this study were defined as family members who assumed the primary caregiving responsibility for an adult ICU patient during hospitalization and who provided unpaid physical care, emotional support, or assistance with medical decision-making throughout the patient’s illness.

Caregivers were eligible for inclusion if they met all of the following criteria: (1) aged 18 years or older; (2) the patient under their care had an ICU stay of at least 48 h, was aged 18 years or older, and remained hospitalized in the same institution after ICU discharge; (3) served as the primary caregiver during the patient’s illness; (4) were able to complete the survey either in written or oral form; and (5) provided signed informed consent.

Caregivers were excluded if any of the following applied: (1) their own physical condition was too poor to participate; (2) a history of severe mental illness, psychiatric disorder, or sleep disorder; (3) the presence of another major organic disease; (4) severe hearing or speech impairment affecting communication; (5) the patient they cared for did not survive during the same hospital stay; or (6) the patient they cared for was a pediatric ICU patient.

### Sample size estimation

Different samples were used for EFA and CFA. Following the guidelines of a 9:1 ratio of participants to items for factor analysis, a sample of 396 was required to analyse the 22 items in both the EFA and the CFA [34]. A total of 468 questionnaires were distributed, accounting for a potential attrition rate of 10–20%. The collected questionnaires were all included in the final analysis.

### Data collection tool

The data collection instruments consisted of two parts. In the first part, demographic and clinical information of ICU patients, such as age and diagnosis, was gathered. Their family caregivers’ demographic information was also gathered. The second part consisted of the SF-36 and the PICS‑F Scale.

### Data collection

Before data collection, research personnel received standardized training on procedures and questionnaire content. The study objectives and data protection measures were carefully explained to obtain informed consent from family caregivers. Assessments were conducted from two to four week after patient ICU transfer until hospital discharge [35]. The questionnaires were distributed in person. For those unable to complete the forms independently, assistance was provided to clarify the items and record the responses. Each questionnaire required approximately 5 to 10 min and was checked onsite for completeness after collection.

### Statistical analysis

SPSS version 25.0 and AMOS version 24.0 (IBM Inc., Armonk, NY) were used to perform the statistical analyses. Descriptive statistics were adopted to illustrate participants’ characteristics. To examine baseline differences of subgroups, chi-square tests were chosen. A level of statistical significance was set at *p* < 0.05. Item analysis was performed by calculating the mean, kurtosis, and item‒item correlations, as well as item‒total correlations for each candidate item. The standardized factor loadings were examined to assess convergent validity. Discriminant validity was also established. The Kaiser–Meyer–Olkin (KMO) measure and Bartlett’s test of sphericity were applied to ensure the factorability of the data. EFA was conducted using principal component analysis with Promax rotation. Items were retained on the basis of the following criteria: communality greater than 0.30, a primary factor loading above 0.40, and no significant cross-loadings, which was defined as having a loading greater than 0.30 on more than one factor with a difference of less than 0.20 [36]. This derived structure was subsequently tested using CFA on an independent subsample. Criterion validity was assessed by analysing the correlations between the PICS-F Scale and the 36-Item Short Form Health Survey questionnaire, known as the SF-36. This analysis was grounded in the established understanding that the PICS-F negatively affects the quality of life of ICU patients’ caregivers. The measurement of their quality of life was conducted using the Chinese version of the SF-36, a tool developed and validated by Li, Wang, and Shen (2002) [37]. This instrument consists of eight subscales, namely, physical functioning (PF), role-physical (RP), bodily pain (BP), general health (GH), vitality (VT), social functioning (SF), role-emotional (RE), and mental health (MH). The Chinese version of the SF-36 demonstrated good overall reliability, with a Cronbach’s α of 0.87 in a validation study [38]. The internal consistency of the PICS-F Scale was also assessed by Cronbach’s α.

### Ethical considerations

#### Ethical approval

(Protocol 2024-H-124) was granted by the Ethics Committee of the Second Affiliated Hospital of Shandong First Medical University on 6 December 2024. Data were anonymized immediately after collection. Personal identifiers were removed and replaced with unique participant codes. The coding list was kept separately in a secure location. All data was stored on a password‑protected computer accessible only to the research team.

## Results

### Results of literature review

The preliminary search retrieved a total of 7569 records. After removing duplicates and excluding articles not associated with the study, 13 articles were finally included for full-text review. Information related to PICS-F symptoms was extracted from these studies and grouped into the four domains of physical, mental, cognitive, and social. The family caregivers of ICU patients experience problems across these four domains, including physical problems, such as sleep disturbances, headaches, loss of appetite, and fatigue [16]; mental health problems, such as anxiety, depression, complicated grief, and PTSD [39]; cognitive problems, such as compromised decision-making ability [17]; and social problems, such as lifestyle disturbances and reduced employment [40]. Based on these symptoms, a preliminary 30-item pool with four distinct dimensions was developed.

### Results of expert consultation

This study selected 18 experts from three general hospitals in Shandong Province. The expert panel included nurses from internal medicine and surgical departments, mental health professionals from psychological practices, and physicians from critical care practices. Seventeen experts finally completed the Delphi consultation round (Table 1).

**Table 1:** General Information of the Consulting Experts (*N* = 17)

Items	Categories^1^	Number	*N* (%)
Sex	Male	4	23.5
	Female	13	76.5
Age	35–45 years	6	35.1
	45–55 years	8	47.2
	> 55 years	3	17.7
Professional level	Chief academic clinical specialist	9	52.9
	Associate chief clinical specialist	6	35.3
	Intermediate clinical specialist	2	11.8
Academic degree	Bachelor’s degree	10	58.8
	Master’s degree	3	17.6
	Doctoral degree	4	23.5
Field of expertise	Critical care	5	29.4
	Psychology	5	29.4
	Nursing	7	41.2

^1^Categories” means the subgroups or levels within each variable

A two-round Delphi consultation was conducted to refine the scale. In the first round, based on screening criteria and expert feedback, 11 items were modified, 9 items were deleted, and 1 item was added, resulting in a provisional structure of 4 dimensions and 22 items. The second round modified 1 item. Finally, the scale was finalized with 4 dimensions and 22 items (Supplementary 1). The consultation process demonstrated high methodological quality. In the first round, 18 questionnaires were sent out and 17 were returned, a response rate of 94.4%. In the second round, 17 were sent out, and all 17 were returned, a response rate of 100%. One expert who did not respond in the first round was not invited to the second round. The experts showed substantial authority, with coefficients of 0.91 and 0.89. Kendall’s W coefficient increased from 0.138 (χ² = 51.698, *p* < 0.01) in the first round to 0.219 (χ² = 108.143, *p* < 0.01) in the final round. Both results achieved statistical significance (*p* < 0.01). The four dimensions met the retention criteria across both rounds, as their mean importance scores and coefficients of variation remained within acceptable thresholds.

### Characteristics of the participants

In the EFA phase, 210 participants’ responses were received. During the CFA phase, 258 participants were invited. Detailed demographic information of both ICU patients (Table 2) and their family caregivers (Table 3) was collected in the two phases. Among the total 468 caregivers, 230 were male (49.1%), and 238 were female (50.9%). The median ICU length of stay for ICU patients was 7 days.

**Table 2:** Characteristics of the patients (*N* = 468)

Characteristics	Categories	Total *n* (%)	EFA group *n* = 210	CFA group *n* = 258	t or χ² (*p*)
Age (yrs)	≤ 40	32 (6.8)	14 (6.7)	18 (7.0)	14.081 (0.001)
	41–59	143 (30.6)	46 (21.9)	97 (37.6)	
	≥ 60	293 (62.6)	150 (71.4)	143 (55.4)	
Sex	Male	266 (56.8)	121 (57.6)	145 (56.2)	0.095 (0.758)
	Female	202 (43.2)	89 (42.4)	113 (43.8)	
Diagnosis	Respiratory diseases	31 (6.6)	16 (7.6)	15 (5.8)	20.519 (0.015)
	Cardiovascular diseases	74 (15.8)	43 (20.5)	31 (12.0)	
	Gastrointestinal diseases	42 (9.0)	19 (9.0)	23 (8.9)	
	Renal and urinary diseases	9 (1.9)	2 (1.0)	7 (2.7)	
	Endocrine and metabolic disorders	7 (1.5)	6 (2.9)	1 (0.4)	
	Haematological diseases	5 (1.1)	1 (0.5)	4 (1.6)	
	Neurological diseases	152 (32.5)	69 (32.9)	83 (32.2)	
	Toxic and physical injuries	4 (0.9)	3 (1.4)	1 (0.4)	
	Surgical diseases	140 (29.9)	50 (23.8)	90 (34.9)	
	Gynaecological and obstetric diseases	4 (0.9)	1 (0.5)	3 (1.2)	
ICU admission days^2^	< 10 days	318 (67.9)	136 (64.8)	182 (70.5)	7.530 (0.023)
	10–20 days	114 (24.4)	50 (23.8)	64 (24.8)	
	> 20 days	36 (7.7)	24 (11.4)	12 (4.7)	
Number of	1	415 (88.7)	186 (88.6)	229 (88.8)	0.086 (0.958)
admissions	2	41 (8.8)	19 (9.0)	22 (8.5)	
	≥ 3	12 (2.6)	5 (2.4)	7 (2.7)	
Educational level	Primary school or below	186 (39.7)	98 (46.7)	88 (34.1)	8.325 (0.040)
	Junior secondary school	160 (34.2)	67 (31.9)	93 (36.0)	
	Secondary or vocational school	98 (20.9)	36 (17.1)	62 (24.0)	
	Associate degree or above	24 (5.1)	9 (4.3)	15 (5.8)	
Occupation	Unemployed	103 (22.0)	28 (13.3)	75 (29.1)	25.057 (0.0001)
	Farming	158 (33.8)	81 (38.6)	77 (29.8)	
	Employee	44 (9.4)	17 (8.1)	27 (10.5)	
	Retired	94 (20.1)	56 (26.7)	38 (14.7)	
	Freelancer	69 (14.7)	28 (13.3)	41 (15.9)	
Use of mechanical ventilation	Yes	324 (69.2)	189 (90.0)	135 (52.3)	77.138 (0.0001)
	No	144 (30.8)	21 (10.0)	123 (47.7)	

^2^Number of admissions means the total hospital admissions per patient up to the time of the survey

**Table 3:** Characteristics of the family caregivers (*N* = 468)

Characteristics	Categories	Total *n* (%)	EFA group *n* = 210	CFA group *n* = 258	t or χ² (*p*)
Age	≤ 40	73 (15.6)	51 (24.3)	22 (8.5)	51.006 (0.0001)
	41–59	249 (53.2)	126 (60.0)	123 (47.7)	
	≥ 60	146 (31.2)	33 (15.7)	113 (43.8)	
Sex	Male	230 (49.1)	113 (53.8)	117 (45.3)	3.316 (0.069)
	Female	238 (50.9)	97 (46.2)	141 (54.7)	
Relationship to the patient	Spouse	315 (67.3)	66 (31.4)	249 (96.5)	225.285(0.0001)
	Child	148 (31.6)	141 (67.1)	7 (2.7)	
	Parent	5 (1.1)	3 (1.4)	2 (0.8)	
Marital status	Married	454 (97.0)	203 (96.7)	251 (97.3)	0.153 (0.926)
	Single	12 (2.6)	6 (2.9)	6 (2.3)	
	Widowed	2 (0.4)	1 (0.5)	1 (0.4)	
Educational level	Primary school or below	112 (23.9)	24 (11.4)	88 (34.1)	35.818(0.0001)
	Junior secondary school	172 (36.8)	82 (39.0)	90 (34.9)	
	Senior secondary school	146 (31.2)	84 (40.0)	62 (24.0)	
	Associate degree or above	38 (8.1)	20 (9.5)	18 (7.0)	

### Item analysis

The item analysis proceeded in three steps. Independent-samples t-tests indicated that items showed differences in scores in the high and low groups (t-values ranged from 9.036 to 17.927, all *p* < 0.001), confirming their discriminant ability. All 22 items demonstrated positive correlations with the total scale score, with coefficients ranging from 0.560 to 0.734 (all *p* < 0.001).

### Validity

EFA was performed on the 22 initial items. The Kaiser–Meyer–Olkin measure confirmed the sampling adequacy (KMO = 0.886), and Bartlett’s test of sphericity suggested that the correlations were appropriate for factor analysis (χ²=4075.656, *p* < 0.001). The scree plot and the eigenvalue-greater-than-one criterion supported a four-factor solution. All 22 items met the predefined retention criteria. The four factors collectively explained 76.33% of the total variance (Table 4). In accordance with the conceptual framework, the factors were labelled the physical, mental, cognitive, and social domains.

**Table 4:** Factor loadings from EFA

Items	Factor				Communality
	1	2	3	4	
5. I feel panicked.	**0.851**	0.107	0.118	0.137	0.768
4. I often have headaches, neck pain, back pain, and lumbago pain.	**0.842**	0.126	0.24	0.127	0.799
1. I feel tired.	**0.840**	0.100	0.083	0.149	0.744
2. My appetite decreases.	**0.804**	0.195	0.138	0.154	0.727
3. My sleep quality declines.	**0.797**	0.191	0.222	0.195	0.759
13. I will recall the scene of him/her entering the ICU.	0.041	**0.910**	0.117	0.112	0.856
9. I feel depressed.	0.131	**0.894**	0.108	0.143	0.848
8. I get angry easily.	0.09	**0.876**	0.161	0.144	0.823
10. I feel hopeless.	0.114	**0.848**	0.151	0.09	0.763
12. I feel self-blame or resentment for his/her poor condition.	0.179	**0.838**	0.082	0.147	0.763
11. I feel lonely and helpless.	0.107	**0.824**	0.18	0.128	0.739
6. I feel agitated and restless.	0.136	**0.813**	0.121	0.124	0.709
7. I feel worried and afraid.	0.162	**0.797**	0.112	0.143	0.694
16. I am less efficient at completing tasks.	0.218	0.165	**0.844**	0.160	0.813
15. I am unable to concentrate.	0.072	0.202	**0.833**	0.202	0.781
17. I have difficulty making decisions.	0.203	0.174	**0.824**	0.162	0.777
14. I’m forgetful.	0.254	0.180	**0.736**	0.167	0.666
19. My work has been affected by caring for him/her.	0.160	0.138	0.103	**0.834**	0.751
18. I feel significant financial pressure.	0.088	0.183	0.082	**0.833**	0.742
20. Caring for him/her takes up my own time.	0.165	0.196	0.165	**0.828**	0.778
21. I participate less in activities I enjoy.	0.190	0.078	0.205	**0.813**	0.745
22. My family conflicts have increased or worsened.	0.157	0.170	0.192	**0.812**	0.750
Eigen value	3.828	6.158	3.038	3.769	
Explained variance (%)	17.400	27.993	13.807	17.130	
Total explained variance (%)	17.400	45.393	59.200	76.330	

CFA was performed to test the four-factor structure identified in the exploratory factor analysis. Model fit was verified against multiple standard indices. The results indicated an acceptable fit to the data. The chi-square statistic was significant, with χ² = 620.940, df = 203, and *p* < 0.001. The degrees of freedom to chi-square ratio was 3.059. The RMR was 0.056, the SRMR 0.075, and the RMSEA 0.090. Furthermore, the Incremental Fit Index (IFI) was 0.928, the Tucker–Lewis index (TLI) reached 0.917, and the comparative fit index (CFI) was 0.927 (Table 5).

**Table 5:** The model’s fit indicators

	χ²	df	Standardized χ²(CMIN/DF)	RMR	SRMR	RMSEA	IFI	TLI	CFI
Criteria	> 0.05		≤ 3.0	≥ 0.05	≥ 0.05	≤ 1	≥ 0.90	≥ 0.90	≥ 0.90
Model	620.940 < 0.001	203	3.059	0.056	0.075	0.090	0.928	0.917	0.927

**Fig. 1:**
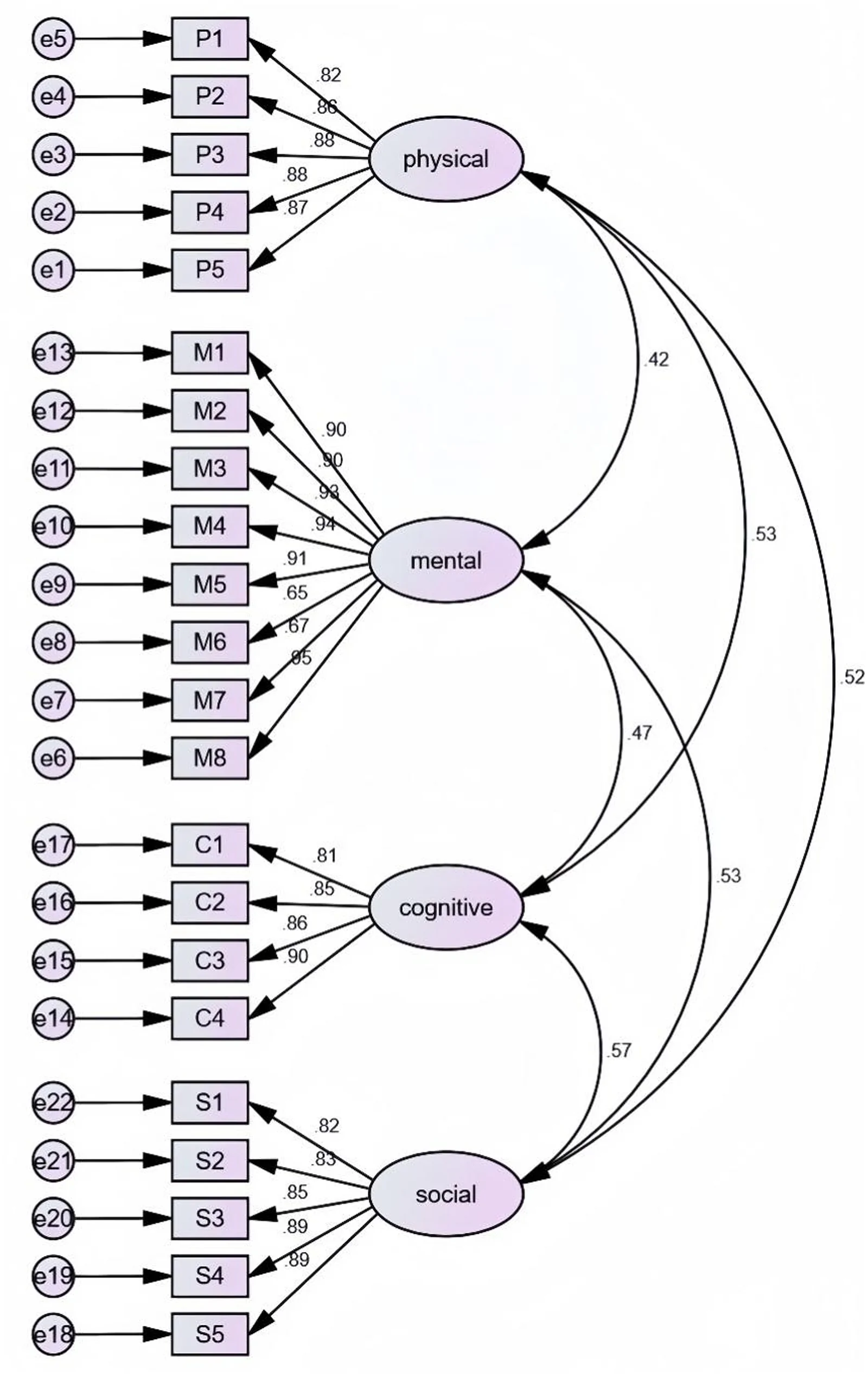
The Standardized Estimates of the CFA Model

To assess convergent validity, the standardized factor loadings were examined. All the items demonstrated loadings above the recommended threshold of 0.50 (Figure. 1). For discriminant validity, the criterion, which requires that the interval of the correlation coefficient between any two factors plus and minus two standard deviations does not include the value of 1, was employed. The analysis confirmed that this criterion was met.

For criterion validity, the total score of the PICS-F Scale was negatively correlated with the total score of the SF-36, with *r* = − 0.723 and *p* < 0.001. Negative correlations were also found between each of the four subscales of the PICS-F and the SF-36 total score, with correlation coefficients from − 0.440 to − 0.600, all significant at *p* < 0.001 (Table 6).

**Table 6:** Correlations between the PICS-F total score and SF-36 subscale scores

		Physical	Mental	Cognitive	Social	PICS-F
SF-36	r	-0.499**	-0.600**	-0.440**	-0.549**	-0.723**
	*p* value	< 0.001	< 0.001	< 0.001	< 0.001	< 0.001

** *p* < 0.01, * *p* < 0.05

### Reliability

Regarding internal consistency, the Cronbach’s α was 0.918 for the physical factor, 0.958 for the mental factor, 0.891 for the cognitive factor, and 0.915 for the social factor. The overall internal consistency for the total scale was 0.934.

### The finalized instrument

The finalized PICS-F Scale comprises 22 items across four domains, namely, physical, mental, cognitive, and social. It uses a 4-point response scale (0 = never to 3 = always), with total scores from 0 to 66 (Table 7).

**Table 7:** Descriptive statistics of scores for the PICS-F scale

Scale	Items	Score Range	Total Score Mean ± SD	Item Mean ± SD	Cronbach’s α
Physical	5	0–15	7.00 ± 4.09	2.80 ± 0.82	0.918
Mental	8	0–24	9.61 ± 6.42	2.75 ± 0.80	0.958
Cognitive	4	0–12	5.49 ± 3.22	3.00 ± 0.81	0.891
Social	5	0–15	6.51 ± 3.96	2.80 ± 0.79	0.915
PICS-F Scale	22	0–66	28.69 ± 13.09	1.30 ± 0.60	0.934

## Discussion

The developed PICS-F Scale provides a useful tool for the full assessment of problems experienced by family caregivers of ICU patients. It also fits the initially established conceptual framework.

In terms of the physical domain, the scale comprises five items, including fatigue, decreased appetite, poor sleep quality, bodily pain, and palpitations, which represent common issues among family caregivers. In a study, caregivers’ physical health dimension scores were initially low at enrolment and showed no significant improvement over the 60-day follow-up period, remaining substantially below the optimal health level throughout. This, in turn, affects caregivers’ overall quality of life [41]. In addition, a cohort study revealed that approximately 77% of family caregivers reported sleep problems after ICU transfer, and 57.6% of caregivers experienced moderate to high levels of fatigue [42]. Previous research used separate instruments. Instead, the PICS-F Scale involves the assessment of a range of physical problems.

Current assessment tools for the mental dimension have their specific strengths, yet each has certain limitations when applied to PICS‑F screening in clinical practice. The Clinician-Administered PTSD Scale for DSM-5 is a diagnostic gold standard due to its structured interview format and high diagnostic accuracy. However, it requires specialized training and significant administration time, which limits its feasibility for large-scale screening or rapid bedside assessment. The Brief Symptom Inventory-18 covers anxiety, depression, and somatization, but its items may not be ICU-related, such as caregiver burden or traumatic memories. This reduces its accuracy in identifying PICS‑F‑specific mental symptoms [43]. The widely used Hospital Anxiety and Depression Scale is mainly developed for anxiety and depression assessment, so it does not assess major PTSD features. Therefore, it may not capture the overall mental symptoms associated with PICS‑F [44]. Other commonly used measures such as the Generalized Anxiety Disorder 7-item and Patient Health Questionnaire-9 are efficient single‑domain tools for specific symptom assessment. Thus, a full evaluation of PICS-F requires multiple instruments and increases assessment cost and respondent burden [45]. In that case, the PICS-F Scale is an appropriate tool to meet the clinical needs for mental assessment.

With respect to the cognitive domain, four items assess forgetfulness, poor concentration, reduced task efficiency, and indecisiveness. Recent PICS‑F research usually operates within a mental‑physical‑social framework that does not formally include the cognitive dimension, despite qualitative evidence indicating cognitive impairment in family caregivers. For instance, in qualitative interviews, caregivers reported having to assist patients with the cognitive sequelae of critical illness while themselves experiencing symptoms such as poor concentration, memory lapses, and decision-making fatigue [26]. This study shows the great need for a validated tool, such as the PICS-F Scale, to reliably measure these issues.

Financial pressure, negative employment impacts, significant time commitment, decreased personal engagement, and increased family conflicts are all included in the social domain. A study of stress management interventions for spouses of ICU patients found that spouses who experienced ICU-related stress had lower levels of perceived emotional support, instrumental support, life satisfaction, and sense of meaning than those in the control group, as well as significantly higher levels of perceived social rejection and hostility. Additionally, 90% of family caregivers reported stress levels above the clinical threshold within 36 h of their family members who were admitted to the ICU. Furthermore, long-term ICU caregiving experience negatively affects caregivers’ social cognition, increasing the risk of interpersonal alienation and conflict [46]. However, current tools frequently measure isolated indicators such as family function or social support, which leads to an incomplete assessment of overall social functioning [47]. Moreover, many available multidimensional instruments are complex and time-consuming in procedures, limiting their suitability for rapid screening or large-scale surveys [48]. The PICS-F Scale aims to address the problem of inconsistent data collection to promote the research progress of PICS-F.

In summary, by integrating the physical, mental, cognitive, and social dimensions into a single instrument, the PICS-F Scale addresses the limitations of current tools and provides a standardized and efficient assessment tool. The PICS-F Scale also has significant practical advantages for implementation. Its final 22-item format reduces the burden on stressed caregivers. The average completion time is approximately 5 to 10 min, making brief clinical encounters possible. The items are written in simple, straightforward language, so family members can complete the scale on their own without the need for specialized guidance. In addition, its straightforward scoring allows healthcare professionals to quickly interpret the results and identify the at-risk family caregivers. If the rapid screening shows a high risk of PICS‑F in a family caregiver, more detailed assessments can then be performed through single‑domain instruments, such as specific mental, cognitive, or social evaluations. In that case, the caregivers can be offered an appropriate intervention in time. Therefore, the PICS-F Scale is a practical tool, enabling rapid screening in research and early identification of at-risk individuals in clinical settings.

### Limitations

This study developed and validated a PICS-F assessment tool, which benefits clinical practice and further research.

The study has several limitations that should be mentioned. First, a convenience sampling method was used to collect samples from general hospitals in Shandong Province. This may affect the general applicability of the results to family caregivers in other geographic locations, cultural contexts, or different types of ICUs. Future studies with more diverse and representative samples are warranted. Second, test–retest reliability could not be calculated in this study. Follow-up data were not collected, mainly because of limited follow-up conditions and clear differences between the setting after discharge and the baseline. As a result, the stability of the scale over time remains untested. This is a major limitation in the current psychometric evidence for the scale. Third, this study employed a cross‑sectional design and assessed caregivers only once before ICU discharge. Follow-up at later time points after discharge, was not included. Therefore, whether the scale can reflect or predict long‑term PICS‑F status requires further investigation. Future longitudinal studies should be conducted to address these problems. It should also be noticed that the PICS‑F Scale was developed and validated only among family caregivers of adult ICU survivors. Therefore, whether the scale is suitable for caregivers of pediatric patients or patients who do not survive during the hospitalization remains unknown and should be examined in future research. Finally, the Cronbach’s α for the mental subscale was 0.958, above the usual 0.95 cutoff. This may be because the items measure closely tied symptoms of the same mental distress, and the family caregivers in our sample responded in a similar way. Although a high α may suggest some item redundancy, a little elevated internal consistency is acceptable for a screening tool, since it helps ensure adequate domain coverage without greatly increasing respondent burden. Future studies should use larger samples to further examine the items and consider whether reduction is warranted.

## Conclusion

The PICS-F Scale is a reliable instrument that can standardize the early assessment of PICS-F in family caregivers of adult ICU survivors in Chinese-language settings. Its use is recommended in the early phase after ICU survivors are transferred out of the ICU to promote further research on PICS-F.

## Supplementary Information

Below is the link to the electronic supplementary material.

Supplementary Material 1

## Data Availability

The datasets of this study are confidential and cannot be available.

## Abbreviations

AMOS: Analysis of Moment Structures
Cr: Authority Coefficient
CFI: Comparative Fit Index
CFA: Confirmatory Factor Analysis
EFA: Exploratory Factor Analysis
IFI: Incremental Fit Index
ICU: Intensive Care Unit
KMO: Kaiser–Meyer–Olkin
PICS: Post-Intensive Care Syndrome
PICS-F: Post-Intensive Care Syndrome-Family
PTSD: Posttraumatic Stress Disorder
RMR: Root Mean Square Residual
RMSEA: Root Mean Squared Error of Approximation
SD: Standard Deviation
SF-36: 36-Item Short Form Health Survey questionnaire
SPSS: Statistical Package for the Social Sciences
SRMR: Standardized Root Mean Square Residual
TLI: Tucker-Lewis Index
X2 /df: Chi-Square/Degrees of Freedom Ratio
