# Supplementary material for: Development and validation of a post-ICU syndrome in family caregivers scale (PICS-F Scale)
Source: BMC Nurs. 2026 Aug 8;25:894. doi: 10.1186/s12912-026-05207-5 (PMC13621639; doi:10.1186/s12912-026-05207-5)
Supplement: Supplementary file 1 [file 12912_2026_5207_MOESM1_ESM.docx]

**Supplementary 1. Final version of the post-intensive care syndrome-family Scale**

| **Items** | **Never** | **Sometimes** | **Most often** | **Always** |
| --- | --- | --- | --- | --- |
| 1. I feel tired. | 0 | 1 | 2 | 3 |
| 2. My appetite decreases. | 0 | 1 | 2 | 3 |
| 3. My sleep quality declines. | 0 | 1 | 2 | 3 |
| 4. I often have headaches, neck pain, back pain, and lumbago pain. | 0 | 1 | 2 | 3 |
| 5. I feel panicked. | 0 | 1 | 2 | 3 |
| 6. I feel agitated and restless. | 0 | 1 | 2 | 3 |
| 7. I feel worried and afraid. | 0 | 1 | 2 | 3 |
| 8. I get angry easily. | 0 | 1 | 2 | 3 |
| 9. I feel depressed. | 0 | 1 | 2 | 3 |
| 10. I feel hopeless. | 0 | 1 | 2 | 3 |
| 11. I feel lonely and helpless. | 0 | 1 | 2 | 3 |
| 12. I feel self-blame or resentment for his/her poor condition. | 0 | 1 | 2 | 3 |
| 13. I will recall the scene of him/her entering the ICU. | 0 | 1 | 2 | 3 |
| 14. I’m forgetful. | 0 | 1 | 2 | 3 |
| 15. I am unable to concentrate. | 0 | 1 | 2 | 3 |
| 16. I am less efficient at completing tasks. | 0 | 1 | 2 | 3 |
| 17. I have difficulty making decisions. | 0 | 1 | 2 | 3 |
| 18. I feel significant financial pressure. | 0 | 1 | 2 | 3 |
| 19. My caregiving responsibilities interfere with my work. | 0 | 1 | 2 | 3 |
| 20. Caring for him/her takes up my own time. | 0 | 1 | 2 | 3 |
| 21. I participate less in activities I enjoy. | 0 | 1 | 2 | 3 |
| 22. My family conflicts have increased or worsened. | 0 | 1 | 2 | 3 |
